# Preoperative weight loss interventions before total hip and knee arthroplasty: a systematic review of randomized controlled trials

**DOI:** 10.1186/s42836-024-00252-4

**Published:** 2024-05-17

**Authors:** Lawrence Chun Man Lau, Ping Keung Chan, Tak Wai David Lui, Siu Wai Choi, Elaine Au, Thomas Leung, Michelle Hilda Luk, Amy Cheung, Henry Fu, Man Hong Cheung, Kwong Yuen Chiu

**Affiliations:** 1 Department of Orthopaedics and Traumatology, School of Clinical Medicine, The University of Hong Kong, Hong Kong SAR, China; 2 Department of Medicine, School of Clinical Medicine, The University of Hong Kong, Hong Kong SAR, China; 3 Department of Orthopaedics and Traumatology, Queen Mary Hospital, Hong Kong SAR, China

**Keywords:** Arthroplasty, Weight loss intervention, Diet modification, Bariatric surgery, Systematic review

## Abstract

**Background:**

The high co-prevalence of obesity and end-stage osteoarthritis requiring arthroplasty, with the former being a risk factor for complications during arthroplasty, has led to increasing interest in employing preoperative weight loss interventions such as bariatric surgery and diet modification. However, the current evidence is conflicting, and this study aimed to investigate the effect of weight loss intervention before arthroplasty in prospective randomized controlled trials.

**Methods:**

Four electronic databases (MEDLINE, EMBASE, Web of Science, and Cochrane Central Register of Controlled Trials) were searched for prospective randomized controlled trials that compared weight loss interventions with usual care from inception to October 2023 by following the PRISMA guidelines. The Cochrane risk of bias tool and GRADE framework were used to assess the quality of the studies. Meta-analyses were performed when sufficient data were available from 2 or more studies.

**Results:**

Three randomized controlled trials involving 198 patients were identified. Two studies employed diet modification, and one study utilized bariatric surgery. All three studies reported significant reductions in body weight and body mass index (BMI), and intervention groups had fewer postoperative complications. There was no difference in the length of stay between the intervention group and the control group. Variable patient-reported outcome measures were used by different research groups.

**Conclusion:**

Weight loss intervention can achieve significant reductions in body weight and body mass index before arthroplasty, with fewer postoperative complications reported. Further studies with different populations could confirm the effect of these interventions among populations with different obesity characteristics.

**Supplementary Information:**

The online version contains supplementary material available at 10.1186/s42836-024-00252-4.

## Introduction

Obesity is becoming a global burden, and disability-adjusted life years are predicted to increase by 39.8% worldwide from 2020 to 2030 [1]. Longitudinal population-based studies have reported that an increase in body weight leads to an increased risk of osteoarthritis (OA) and subsequent risk of arthroplasty [2]. It is also projected that ≥ 69% of primary total knee arthroplasty (TKA) will be performed on obese/morbidly obese patients by 2029 [3]. Obesity is strongly linked to the pathogenesis of OA through its association with chronic inflammation, adipocytokines, and metabolic and mechanical factors [4]. Among the various risk factors, obesity-related mechanical stress was suggested to be the most important risk factor for OA. Since obesity is a known risk factor for perioperative and long-term complications of arthroplasty and is associated with increased risks of pulmonary embolism, superficial infection, periprosthetic joint infection, longer length of stay, wound dehiscence, readmission, reoperation, aseptic loosening, etc. [5–8], weight loss interventions before arthroplasty, through surgical or conservative means, are being increasingly explored to mitigate the risk of obesity during arthroplasty [9, 10]. However, there is conflicting evidence concerning whether these interventions can improve arthroplasty outcomes, as studies suggest that reducing or increasing complications have been reported [10, 11]. Notably, these differences appear to be pronounced between prospective randomized controlled trials and cohort/database studies, particularly cohort studies, which tend to report negative results [9–12]. This could be attributed to several factors. It has been reported that patients with a high body mass index (BMI) and comorbidities, compared to those without comorbidities, are more likely to be provided with weight loss information and be referred for bariatric surgery [13–15]. This means that those who were prescribed weight loss interventions may be in worse overall condition than those not prescribed interventions from the beginning of the study in cohort/database studies; therefore, these two groups were not comparable at baseline [15]. These comorbidities, for example, diabetes and hypoalbuminemia, which are commonly associated with obesity, could also lead to worse outcomes in arthroplasty patients and confound the association between weight loss intervention and arthroplasty outcomes [16]. In addition, weight loss reduces the subsequent incidence of arthroplasty, and those who require arthroplasty in cohort/database studies may be more severe than those who do not [17]. Selection biases were not addressed by previous systematic reviews, and examining only randomized control trials could avoid these biases [11, 12, 18]. These systematic reviews did not examine all modalities of weight loss interventions together either; therefore, the effect of weight loss before arthroplasty had not been completely assessed. Therefore, in this study, we systematically reviewed all randomized controlled trials focusing on patients who underwent arthroplasty for hip and knee osteoarthritis and who underwent weight loss interventions (both surgical and non-surgical) compared to those in the control group without interventions to examine the effect of these weight loss interventions on arthroplasty outcomes and weight control in the postoperative period.

## Methods

### Search strategy

The study followed the Preferred Reporting Items for Systematic Reviews and Meta-Analyses (PRISMA) checklist [19]. The following four databases were used: MEDLINE, EMBASE (Ovid), Web of Science Core Collection (Clarivate), and Cochrane Central Register of Controlled Trials (Wiley). The databases were systematically searched and reviewed by two independent reviewers on October 2023 using Medical Subject Headings (MeSH) and keywords relating to arthroplasty (including total knee arthroplasty, TKA, total knee replacement, total hip arthroplasty, THA, total hip replacement, arthroplasty), osteoarthritis and weight control intervention (including bariatric surgery, weight reduction program, diet, weight loss): (1. Weight reduction program.mp. or exp Weight Reduction Programs/; 2. Bariatric.mp. or exp Obesity, Morbid/or exp Gastroplasty/or exp Bariatrics/or exp Gastric Bypass/or exp Obesity/; 3. Weight loss drug.mp. or exp Anti-Obesity Agents/; 4. Weight loss diet.mp. or exp Diet, Reducing/; 5. Weight loss.mp. or exp Weight Loss/; 6. Pharmacological weight loss.mp.; 7. 1 or 2 or 3 or 4 or 5 or 6; 8. Exp Arthroplasty, Replacement, Knee or exp Arthroplasty, Replacement or exp Arthroplasty or arthroplasty.mp. or exp Arthroplasty, Replacement, Hip/; 9. 7 and 8). The reference lists of all included studies and relevant reviews were also searched for additional publications. This systematic review was registered a priori on PROSPERO (CRD42023472000). There were no restrictions on language or publication date.

### Eligibility criteria

We included prospective randomized controlled trials involving non-obese patients who had undergone either weight control intervention or a control modality prior to knee or hip arthroplasty. Details on the eligibility criteria are described in Table 1. Clinical outcomes after arthroplasty, including medical or surgical complications and weight change, should be reported. Reviews, letters to the editor, case reports, comments, reviews, biochemical studies, conference abstracts, cohort studies, and database studies were excluded.

**Table 1:** Eligibility criteria for inclusion of studies

Parameter	Inclusion criteria	Exclusion criteria
Participants	• Adults aged ≥ 18 years • Overweight and/or obesity • Awaiting elective arthroplasty surgery	• Undergoing non-arthroplasty surgery
Intervention	• Weight‐loss intervention, including (but not limited to): dietary modification, caloric restriction, meal replacement, medication, bariatric surgery)	• No well-defined weight-loss intervention reported
Comparator	• participants underwent usual or standard care	• Control groups that prescribed specific preoperative weight‐loss interventions were excluded
Outcomes	Primary outcomes • Surgical outcomes (patient-reported outcome and postoperative complications) • Secondary outcomes • Body weight change • Body mass index change • Acute length of hospital stay	
Study design	• Prospective randomized controlled trials	• Retrospective studies • Studies retrospectively assessing registry data • Cohort or database studies

### Study selection and data extraction

Two reviewers independently read titles, abstracts, and full texts to assess the eligibility of each study and resolved discrepancies by discussion with a third senior reviewer. The two reviewers used a standardized form to independently extract data on title, author, publication year, study design, patient demographics, weight loss intervention modality, control, outcome, and other variable data from the included papers and compared the results with each other to ensure consensus. Differences among reviewers were resolved through repeated review of the original article and discussion within the team. All analyses were performed using intention-to-treat data.

### Risk of bias assessment

The Cochrane Handbook’s Risk of Bias (RoB) Version 2 checklist was used for quality assessment of each individual outcome from the RCTs based on the five domains of bias (randomization process, deviations from intended interventions, missing outcome data, measurement of the outcome, and selection of the reported result) [20]. Two reviewers assessed the methodological validity of the checklist, and disagreements were resolved through discussion.

### Quality of evidence

The Grades of Recommendations, Assessment, Development and Evaluation (GRADE) framework was utilized to assess the quality of evidence for outcomes [21]. The evidence level could decrease due to study limitations, inconsistency, indirectness, imprecision, and publication bias. Study findings with moderate or large effect sizes could lead to an upgrade of the quality of evidence. Four levels of quality were reported: high, moderate, low, and very low. A summary of the GRADE criteria is presented in Table 2.

**Table 2:** GRADE summary of findings

Outcome	Absolute effect (95% CI)	Relative effect (95% CI)	Participants (studies)	Certainty of the evidence (GRADE)	Comments
Length of stay	Mean difference 0.4 day fewer (1.69 fewer to 0.89 more)	/	198 (3 RCTs)	⨁⨁⨁⨁ High	little to no difference in LOS between both groups.
Surgical outcome (complication free)	115 more per 1,000 (from 13 to 167 more)	OR 2.49 (1.08 to 5.74)	198 (3 RCTs)	⨁⨁◯◯ Low	Weight‐loss intervention may result in surgery without complications but the evidence is uncertain.
Weight change (kg)	Mean difference 10.19 kg lower (17.89 lower to 2.5 lower)	/	198 (3 RCTs)	⨁⨁⨁◯ Moderate	Moderate evidence suggested weight‐loss intervention may result in weight loss during study period.
BMI change	Mean difference 3.45 lower (6.39 lower to 0.5 lower)	/	198 (3 RCTs)	⨁⨁⨁◯ Moderate	Moderate evidence suggested weight‐loss intervention may reduce BMI during study period.

Certainty of the evidence (GRADE):

⨁⨁◯◯ = Low, confidence in the effect estimate is limited; the true effect may be substantially different from the estimate of the effect

⨁⨁⨁◯ = Moderate, moderately confident in the effect estimate, the true effect is likely to be close to the estimate of the effect but there is a possibility that it is substantially different

⨁⨁⨁⨁ = High, very confident that the true effect lies close to that of the estimate of the effect

### Data synthesis and analysis

Meta-analyses were performed when sufficient data existed from 2 or more studies. The heterogeneity of the included studies was assessed by the I^2^ statistic for statistical heterogeneity within each meta-analysis [22]. The I^2^ index was employed to assess statistical heterogeneity among the pooled outcomes, and values were interpreted as follows: low, ≤ 50%; moderate, 51%–74%; and large, ≥ 75% [22]. Odds ratios (ORs) for categorical data and mean differences (MDs) for continuous outcomes with 95% confidence intervals (CIs) were calculated. All the outcomes were pooled on a random-effects model. Sensitivity analysis was not performed due to the small number of studies included. We checked for publication bias using funnel plots. All the statistical analyses were performed using SPSS (IBM SPSS Statistics for Windows, Version 28.0., Released 2021, IBM Corp., Armonk, NY, USA).

## Results

### Study inclusion

**Fig. 1:**
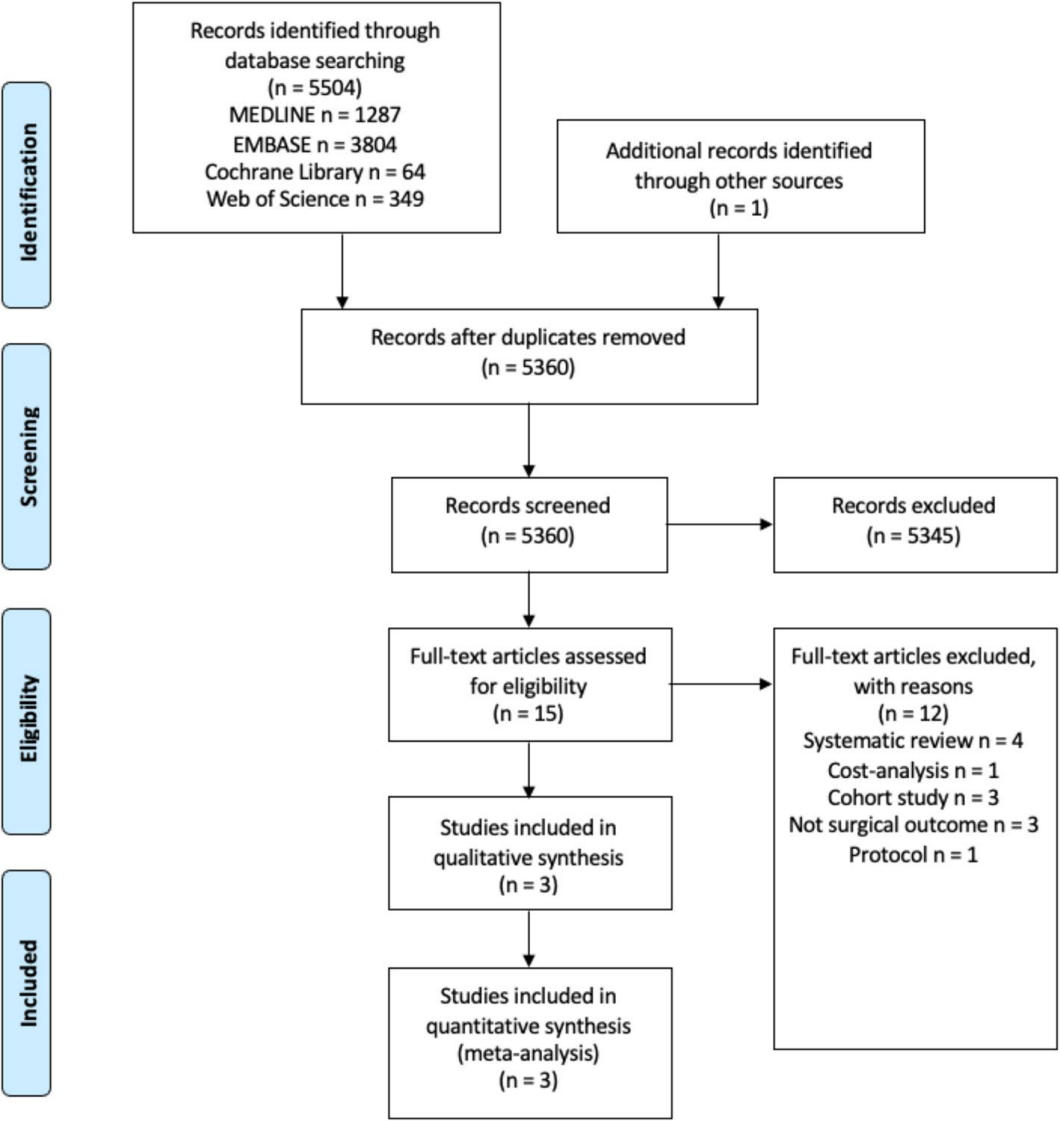
PRISMA flow diagram

A total of 5,504 references were retrieved through an electronic database search with another reference from another source by reviewing the citation list. After excluding irrelevant and duplicated articles whose titles and abstracts were reviewed, 15 additional articles were selected (Fig. 1). Twelve articles were excluded after full text review for the reasons listed in Fig. 1. Three RCTs met the inclusion criteria and provided quantitative data for analysis [9, 10, 23].

### Methodological quality

**Fig. 2:**
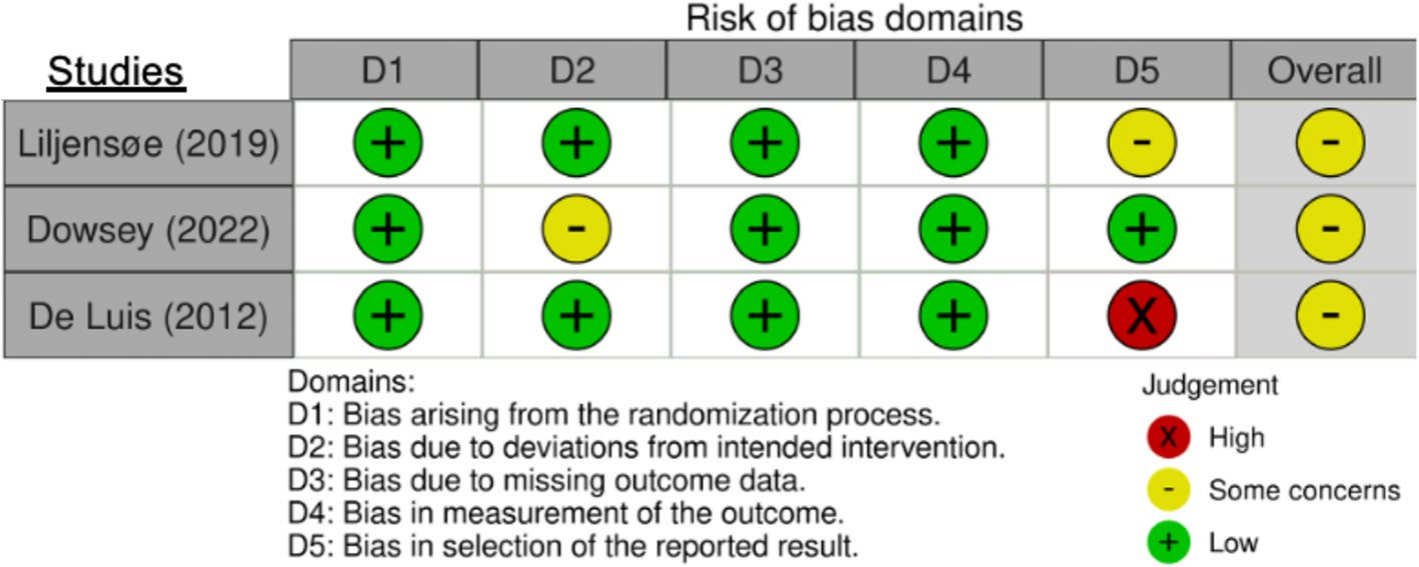
Risk-of-bias assessments for length of hospital stay, surgical outcomes (complication free), weight change, and BMI change

Risk-of-bias assessments were completed for four outcomes: length of stay (LOS), weight change, body mass index (BMI) change, and surgical outcomes (complication free), as shown in Fig. 2. One study demonstrated some concern across all outcomes, with bias due to deviations from the intended intervention. One study included length of stay, weight change, body mass index (BMI) change and high risk of surgical outcomes (complication free) due to selective reporting of outcomes. One study had a high risk of bias for weight change, body mass index (BMI) change and surgical outcome (complication free) due to selective reporting of outcomes.

### Characteristics of the included studies

The three studies included were RCTs published in English. Two studies involved participants with diet control and one participant who underwent bariatric surgery as a weight loss intervention. Two studies involved participants who underwent total knee arthroplasty, and one study involved participants who underwent both total knee arthroplasty and total hip arthroplasty. A total of 198 participants were recruited, 99 and 99 participants were randomized to the control and intervention groups, respectively, with a mean baseline BMI greater than 30 kg/m^2^. Each study focused on different primary and secondary outcomes, with differences occurring during the study periods. The characteristics of the studies included are summarized in Table 3.

**Table 3:** Characteristics of the included studies

Study	Sample size (I/C)	Patient age, mean ± SD, y	Female ratio (%)	Baseline BMI	Intervention duration, m (I/C)	Intervention	Control
Liljensøe et al., 2019, Denmark [9]	38/38	65 (range 46–81)	71%	31.6 (95% CI 30.6–32.6)	2/2	Low-calorie (810 kcal/day) commercial formula and nutritional education	None (none of any nutritional instructions)
de Luis et al., 2012, Spain [23]	20/20	65.0 ± 8.5	82.5%	38.6 ± 4.7	3/3	Hypocaloric commercial formula 1,109.3 kcal/day for lunch and dinner	Dietary advice: reduce 500 cal/day
Dowsey et al., 2022, Australia [10]	41/41	57.8 ± 4.9	80%	43.8 ± 5.5	17/4	Laparoscopic adjustable gastric banding	General weight management advice

*I* Intervention, *C* Control, *SD* Standard deviation, *BMI* body mass index, *y* year, *m* month, *kcal* kilocalorie, *cal* calorie

### Review findings

The types of primary and secondary outcomes are described in Table 4. A meta-analysis was performed where comparable data were available in the included studies, which included the following steps.

**Table 4:** Outcome assessment of the included studies

Study	Primary outcome	Secondary outcome
Liljensøe et al. 2019, Denmark [9]	Short-form 36 subscale Physical Component Score (PCS); body weight and body mass index.	Short-form 36 subscale Mental Component Score (MCS); Knee Injury and Osteoarthritis Outcome Score (KOOS); 6-Minute walk test; body fat mass; bone mineral density; lipid profile; length of stay; intraoperative time; postoperative complications and vital signs
de Luis et al. 2012, Spain [23]	Body weight and BMI; body fat mass; waist circumference.	Lipid profile; insulin resistance; dietary parameters (carbohydrate, saturated fat, poly-unsaturated fat, mono-unsaturated fat); operation duration; length of stay; postoperative complications; haemoglobin change; vital signs and days till independent walking
Dowsey et al. 2022, Australia [10]	Composite: death, perioperative complications, prosthetic infection, and unplanned procedures and/or readmission.	Length of stay; body weight and BMI; pain, function, and QoL using the Western Ontario McMaster Universities Osteoarthritis Index and the Veterans Rand 12 item questionnaires.

#### Length of stay

**Fig. 3:**
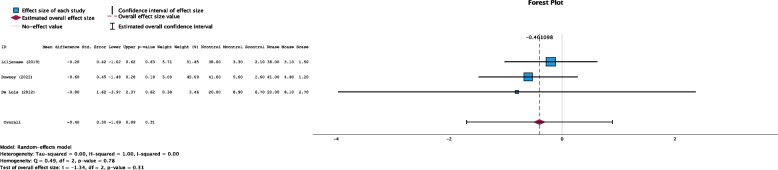
Forest plot showing the pooled standardized effect size with 95% CI for length of stay. For each study, the shaded square represents the point estimate of the intervention effect. The horizontal line joins the lower and upper limits of the 95% CI of these effects. The shaded square area reflects the study’s relative weight in the respective meta-analysis. The diamond at the bottom of the graph represents the estimated overall effect size with the 95% CI for the three study groups

In all three studies, the acute hospital length of stay (LOS) was reported, comprising data from 198 participants (Fig. 3). The pooled effect estimate suggested that the LOS was slightly shorter in the weight loss intervention group than in the control group, but this difference did not reach statistical significance (mean difference −0.40 days, 95% CI: −1.69 to 0.89 days, *P* = 0.31, Funnel plot Fig. S1).

#### Weight change

**Fig. 4:**
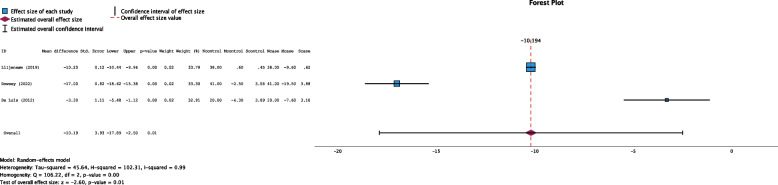
Forest plot showing the pooled standardized effect size with 95% CI for weight change. For each study, the shaded square represents the point estimate of the intervention effect. The horizontal line joins the lower and upper limits of the 95% CI of these effects. The shaded square area reflects the study’s relative weight in the respective meta-analysis. The diamond at the bottom of the graph represents the estimated overall effect size with the 95% CI for the three study groups

All the studies reported mean changes in weight during their study period (Fig. 4). The pooled effect estimate showed low-quality evidence of a statistically significant difference compared with that of the weight loss intervention group, in which more weight loss was achieved during the study period (mean difference −10.19 kg, 95% CI: −17.89 to −2.5 kg, *P* = 0.01, I^2^ = 99%, Funnel plot Fig. S2). There was high heterogeneity among the studies, as they employed different weight loss interventions during different study periods.

#### BMI change

**Fig. 5:**
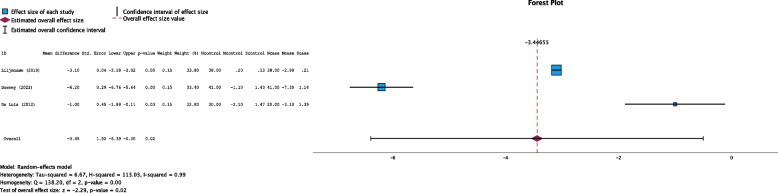
Forest plot showing the pooled standardized effect size with 95% CI for BMI change. For each study, the shaded square represents the point estimate of the intervention effect. The horizontal line joins the lower and upper limits of the 95% CI of these effects. The shaded square area reflects the study’s relative weight in the respective meta-analysis. The diamond at the bottom of the graph represents the estimated overall effect size with the 95% CI for the three study groups

All the studies reported mean changes in BMI during their study period (Fig. 5). The pooled effect estimate showed low-quality evidence of a statistically significant difference, with the weight loss intervention group demonstrating a greater reduction in BMI during the study period (mean difference −3.45, 95% CI: −6.39 to −0.5, *P* = 0.02, I^2^ = 99%, Funnel plot Fig. S3). There was a high heterogeneity among the studies, as they employed different weight loss interventions during different study periods.

#### Operation outcomes – complication free

**Fig. 6:**
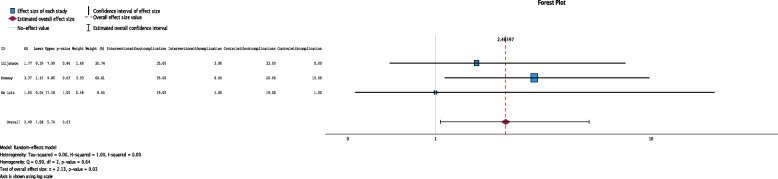
Forest plot showing the pooled standardized effect size with 95% CI for surgery without complications. For each study, the shaded square represents the point estimate of the intervention effect. The horizontal line joins the lower and upper limits of the 95% CI of these effects. The shaded square area reflects the study’s relative weight in the respective meta-analysis. The diamond at the bottom of the graph represents the estimated overall effect size with the 95% CI for the three study groups

In all three studies, the numbers of surgeries that subsequently had and did not cause complications during the study period were reported, comprising data from 198 participants (Fig. 6). The pooled effect estimate suggested that the weight loss intervention group had a significantly greater odds ratio of having no complications after surgery than the control group did (odds ratio 2.49, 95% CI 1.08 to 5.74; *P* = 0.03; I^2^ = 0%, Funnel plot Fig. S4).

#### Patient-reported outcome measures

Two studies reported patient-reported outcome measures (PROMs) with a follow-up period of one year [9, 10]. Liljensøe et al. reported no differences between groups from baseline to 1 year in terms of Short-Form 36 scores (physical component score: 1.3, 95% CI: 2.2 to 4.7; mental component score: 3.3, 95% CI: 0.9 to 7.6) or Knee Injury and Osteoarthritis Outcome Score (activities of daily living: 2.8, 95% CI: −5.8 to 11.4; quality of life: 8.3, 95% CI: −3.4 to 20; symptoms: 4.9, 95% CI: −3.1 to 12.9; pain: 0.8, 95% CI: −9.0 to 10.5; and sports/recreation status: 5.8, 95% CI: −5.5 to 17.1). Dowsey et al. also reported no difference between the two groups from baseline to 1 year in terms of the Western Ontario and McMaster Universities Osteoarthritis Index (OOA) score (pain: 0.6, 95% CI: −9.6 to 10.9; function: −4.7, 95% CI: −12.6 to 3.1; stiffness: −6.5, 95% CI: −16.1 to 3.1; global: −5.0, 95% CI: −13.1 to 3.1); or Veterans Rand 12-item Health Questionnaire (physical component: 3.8, 95% CI: −0.8 to 8.6; mental component: 4.0, 95% CI: −1.4 to 9.4). Since different PROMs were reported, they were not combined in the meta-analysis. De Luis et al. did not report patient-reported outcome measures in their cohort.

## Discussion

In our systematic review, we found that there was a limited amount of high-quality research investigating the effect of weight loss intervention before arthroplasty. Currently, there were only three RCTs that compared weight loss intervention before arthroplasty with conventional care. Among the three weight loss interventions, two were based on diet modification, and one was based on bariatric surgery. The sample size of each study ranged from 40 to 82. Importantly, by pooling the results of three randomized controlled trials, we found that all three of the studies achieved significant weight loss during the study period and that weight loss intervention groups had fewer surgeries with postoperative complications, which has not been reported by previous systematic reviews [11, 12]. There was no difference in the length of stay between the weight loss intervention group and the control group. Due to the different patient-reported outcome measures used by different research groups, their results cannot be combined. Given that an increasing number of knee and hip arthroplasties are being performed on obese/morbidly-obese patients, this study highlights the feasibility and benefit of using the waiting time before arthroplasty to perform weight loss interventions to achieve body weight control and reduce complications. Weight loss interventions also provide additional benefits to middle-aged to elderly patients, such as lowering cardiovascular mortality and all-cause mortality [24].

The strength of this study is that it included randomized controlled trials so that selection bias was minimized, and we could compare the effect of weight loss interventions between comparable groups of patients. Another strength is that all weight loss intervention modalities were considered in this systematic review, thereby providing a meaningful summary examining the effect of weight loss on subsequent arthroplasty surgery and comparing whether all these studies derived similar results with different modalities. Despite the variability of weight loss intervention modalities, all three studies achieved satisfactory body weight loss and a reduction in BMI. By pooling the results, we found that there was a mean reduction of 10.19 kg in body weight and 3.45 in BMI. As expected, the degree of change was greater in patients who underwent bariatric surgery than in those who underwent diet modification, with a magnitude of 1.5 to 2 times greater. Compared to the large number of patients who underwent joint replacement, the representativeness of the three articles was limited because the total sample size was relatively small. This study has several limitations in terms of obtaining highly generalizable results, which are attributed to the currently limited evidence regarding weight loss intervention during arthroplasty, as described below. First, the current study illustrated the limited modalities used for weight loss intervention before arthroplasty. Currently, for example, no exercise or pharmacologically-based interventions have been reported. The modalities that have been reported included weight loss through different diet modifications and bariatric surgery. Although they all aimed to achieve weight loss, the modalities used differed in nature, which introduced heterogeneity and bias to our study. Therefore, the findings should be interpreted by taking this heterogeneity into account. In addition, all the studies were conducted in Caucasian participants with a BMI ranging from 31.6 (class I obesity) to 43.8 (class III obesity), and no Asian studies have investigated the effect of weight loss intervention before arthroplasty. This is particularly important because According to the World Health Organization (WHO), the definitions of overweight and obesity of Asians are lower (elevated BMI ≥ 23 kg/m^2^ and ≥ 25 kg/m^2^, respectively), and Asia has the largest number of obese individuals worldwide [25, 26]. The current study highlights a gap in the body of related knowledge.

Second, this study detected a trend toward a shorter length of stay ( −0.40 days, 95% CI: −1.69 to 0.89 days) but the trend was not statistically significant. Some of the included studies were conducted before the era of enhanced recovery after surgery (ERAS), a multimodal practice that aims at improving postoperative recovery and shortening length of stay and was popularized internationally. Therefore, in current practice, with ERAS, a greater likelihood of a shorter length of stay will increase the difference between the two groups. In particular, Liljensøe et al. reported that more patients (32 patients, 84%) in the diet group than in the control group (24 patients, 63%) were able to mobilize on the day of surgery (day 0) [9]. The same-day mobilization was not reported by the other two studies, but it is a key component of modern ERAS practice, and the effect of weight loss intervention warrants further investigation.

Third, in this study, we found that, compared to those in the control group, the weight loss intervention groups had fewer complications, which was the primary outcome measure (a composite of complications after surgery) used by Dowsey et al. [10]. In the study by Dowsey et al., the greater number of complications in the control group (vs. intervention group) was attributed to the following factors: 8 (vs. 1), 3 (vs. 0) and 3 (vs. 1) knee stiffness [10]. It is important to consider that, in Dowsey et al.’s study, 12 patients in the intervention group declined TKA due to symptomatic relief after weight loss, whereas 2 patients in the usual group declined TKA [10]. This could contribute to a lower number of complications in the intervention group. However, a similar trend was also noted in the study by Liljensøe et al., which included no patients with a declining history of TKA and more complications in the control group (vs. the intervention group): 3 (vs. 1) had wound complications/infections, and 1 (vs. 1) had knee stiffness, while delirium was not mentioned in the study [9]. However, the study by Liljensøe et al. did not intend to use power for the analysis of complications, unlike the Dowsey et al. study. De Luis et al. reported that complications were similar in both groups, but the types of complications and the period during which complications were measured were limited compared to those of the other two studies, which led to a decrease in the overall evidence in view of the risk of bias in reporting. Overall, this study provided evidence that weight loss intervention may contribute to fewer complications after arthroplasty, although this finding needs to be interpreted with caution due to limitations in primary studies, and further RCTs with larger sample sizes could provide further clarification. From a mechanistic point of view, obesity is associated with chronic low-grade systemic inflammation, and weight loss interventions such as bariatric surgery have been reported to lead to resolution of inflammation [27, 28]. This could also explain the lower incidence of complications, especially wounds, after weight loss intervention.

Fourth, since the patient-reported outcomes measured by Dowsey et al. (Western Ontario and McMaster Universities Osteoarthritis Index score and Veterans Rand 12 item Health Questionnaire score) and Liljensøe et al. (Short-Form 36 and Knee injury and Osteoarthritis Outcome Score) were different, they could not be combined, but both reported no difference between the intervention and control groups. However, according to the findings of Dowsey et al., more patients in the intervention group declined TKA due to symptomatic relief after weight loss, and this finding provided indirect concrete evidence that weight loss intervention can improve osteoarthritic patients’ symptomatology and reduce the need for replacement, as did previous publications [17, 29]. In addition, Liljensøe et al.’s study included 7 fewer participants than planned, which might introduce a type 2 error in the outcome and underestimate the trend toward a better Short-Form 36 outcome after weight loss [9]. It has also been reported that a higher BMI is associated with greater pre- to postoperative improvements in outcome scores, as patients are more debilitated before surgery. This may mask the effect of weight loss intervention on patient-reported outcomes after arthroplasty [30].

A conjoint clinical practice guideline was issued in 2023 by the American College of Rheumatology and American Association of Hip and Knee Surgeons regarding the optimal timing of arthroplasty for symptomatic arthritis that failed conservative management. For patients with a BMI of 35–39, 40–49 or ≥ 50, arthroplasty should be performed without delay in achieving weight reduction to a BMI < 35, < 40 or < 50, respectively, based on the evidence of the very low certainty. One study found that these guidelines were derived from a series of observational studies but not from the three RCTs included in the present study [31]. This might be because the guidelines were synthesized before the publication of Dowsey et al.’ study, and the average BMI in Liljensøe et al. was lower than that reviewed by the guidelines. Nevertheless, the findings of the present study support the reviewer’s suggestion that a greater BMI in arthroplasty patients is associated with greater medical and surgical risks, particularly periprosthetic joint infection, and that individuals should be strongly encouraged to reduce weight prior to arthroplasty to mitigate such risk. The current study supplements the guidelines in that a period of weight loss intervention improves patients’ weight (certainty of evidence—moderate), BMI (certainty of evidence—moderate) and surgical outcome with fewer complications (certainty of evidence—low) [27, 28].

## Conclusion

In summary, the potential of weight loss intervention before arthroplasty has been reported in a limited number of randomized controlled trials, and further studies are warranted to investigate its efficacy among different populations with different obesity characteristics.

### Supplementary Information

**Additional file 1: Fig. S1.** Funnel plot for length of stay. **Fig. S2.** Funnel plot for weight changes. **Fig. S3.** Funnel plot for BMI change. **Fig. S4.** Funnel plot for surgical outcome (complication free).

## Data Availability

The datasets used and/or analyzed during the current study are available from the corresponding author upon reasonable request.

## Abbreviations

PROM: Patient-reported outcome measures
TKA: Total knee arthroplasty
THA: Total hip arthroplasty
BMI: Body mass index
CI: Confidence interval
ERAS: Enhanced recovery after surgery
OR: Odds ratio
MD: Mean difference
LOS: Length of stay
