# Supplementary material for: Preoperative weight loss interventions before total hip and knee arthroplasty: a systematic review of randomized controlled trials
Source: Arthroplasty. 2024 May 17;6:30. doi: 10.1186/s42836-024-00252-4 (PMC11100102; doi:10.1186/s42836-024-00252-4)
Supplement: Supplementary file 1 — Additional file 1: Fig. S1. Funnel plot for length of stay. Fig. S2. Funnel plot for weight changes. Fig. S3. Funnel plot for BMI change. Fig. S4. Funnel plot for surgical outcome (complication free). [file 42836_2024_252_MOESM1_ESM.docx]

**Supplementary Information**


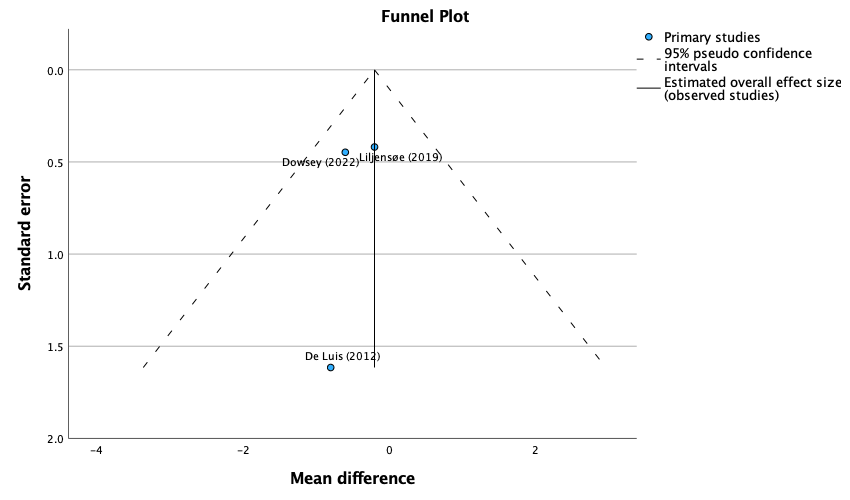


**Fig. S1** Funnel plot for length of stay.


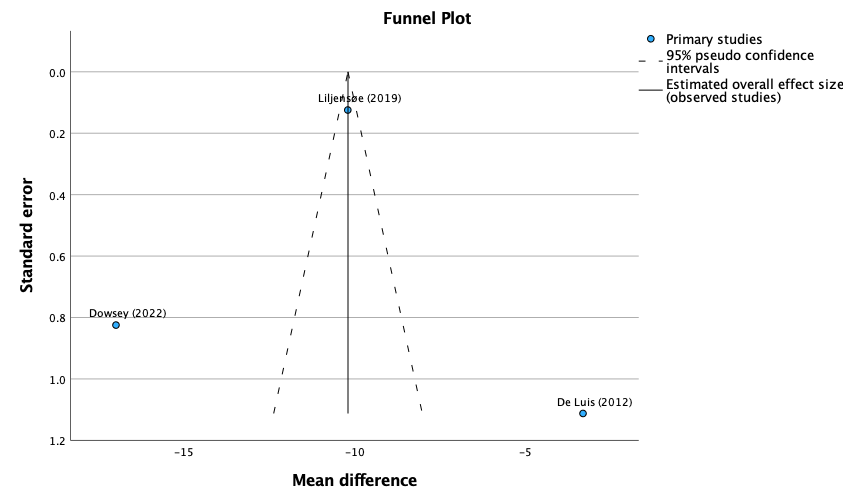


**Fig. S2** Funnel plot for weight changes


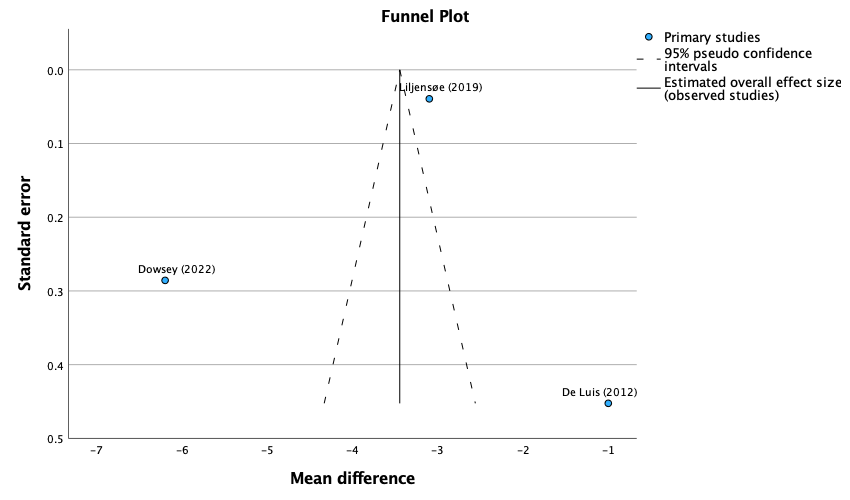


**Fig. S3** Funnel plot for BMI change.


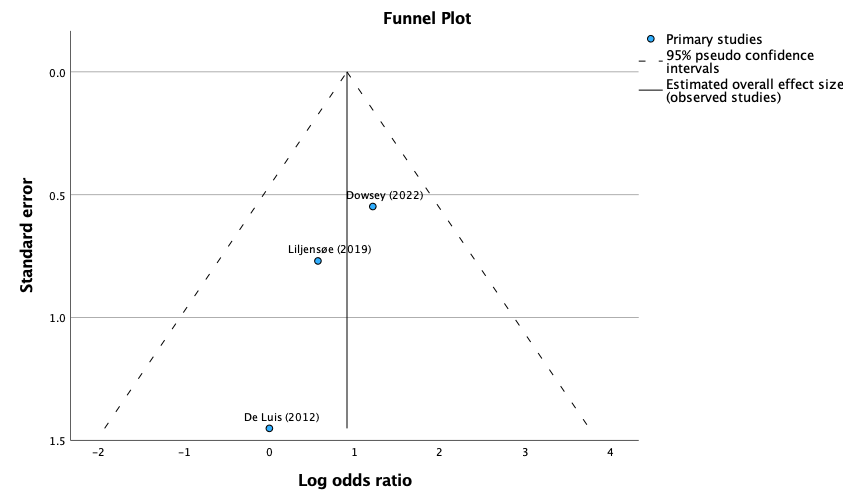


**Fig. S4** Funnel plot for surgical outcome (complication free).
